# Dr. Charles Francis Kiersted

**Published:** 1914-06

**Authors:** 


Dr. Charles Francis Kiersted, Geneva, 1872; died at his home in Gillett, Pa., April 24, aged 69, of heart disease.

